# Biosafety research for non-target organism risk assessment of RNAi-based GE plants

**DOI:** 10.3389/fpls.2015.00958

**Published:** 2015-11-06

**Authors:** Andrew F. Roberts, Yann Devos, Godwin N. Y. Lemgo, Xuguo Zhou

**Affiliations:** ^1^ILSI Research Foundation, Center for Environmental Risk AssessmentWashington, DC, USA; ^2^GMO Unit, European Food Safety AuthorityParma, Italy; ^3^NEPAD Agency – African Biosafety Network of ExpertiseOuagadougou, Burkina Faso; ^4^Department of Entomology, College of Agriculture, Food and Environment, University of KentuckyLexington, KY, USA

**Keywords:** RNAi, environmental risk assessment, plant biotechnology, biosafety research, non-target organisms

## Abstract

RNA interference, or RNAi, refers to a set of biological processes that make use of conserved cellular machinery to silence genes. Although there are several variations in the source and mechanism, they are all triggered by double stranded RNA (dsRNA) which is processed by a protein complex into small, single stranded RNA, referred to as small interfering RNAs (siRNA) with complementarity to sequences in genes targeted for silencing. The use of the RNAi mechanism to develop new traits in plants has fueled a discussion about the environmental safety of the technology for these applications, and this was the subject of a symposium session at the 13th ISBGMO in Cape Town, South Africa. This paper continues that discussion by proposing research areas that may be beneficial for future environmental risk assessments of RNAi-based genetically modified plants, with a particular focus on non-target organism assessment.

## Introduction

The term RNA interference, or RNAi, refers to a collection of biological processes making use of conserved cellular machinery to silence the expression of genes (Hannon, 2002; Mello and Conte, 2004). There are several variations which differ in the source of the RNA and the specific mechanism through which gene silencing is accomplished, but they all are triggered by the presence of a double-stranded RNA (dsRNA) molecule and follow a similar order of events. The dsRNA is processed into small interfering RNAs (siRNAs; normally around 21–25 bp in length) by the protein Dicer or its homologs (RNases) and incorporated into a protein complex known as RISC—the RNA Induced Silencing Complex (Elbashir et al., 2001; Tijsterman and Plasterk, 2004). This complex then uses the siRNA as a template to find and bind to a complementary sequence on a specific messenger RNA (mRNA). Some mismatch in the sequence is allowed (Du, 2005), and the binding of RISC leads to either the degradation of mRNA or the interruption of mRNA translation into protein.

The discovery of the RNAi mechanism did not occur with a single event or publication. The phenomenon had been observed in plants [known as Post Transcriptional Gene Silencing (PTGS), or co-suppression] and fungi (as quelling; Vance and Vaucheret, 2001; Mello and Conte, 2004). The observation that specific and more robust gene silencing could be achieved using dsRNA (rather than single stranded RNA) in a model organism, *Caenorhabditis elegans*, led to the investigation and elucidation of the RNAi machinery as we now understand it (Fire et al., 1998). The initial discovery of the potency of dsRNA as an elicitor of gene silencing was quickly followed by the finding that, at least in *C. elegans*, dsRNA from the environment (in this case produced in *Escherichia coli* bacteria used as a food source) could also trigger gene specific silencing (Timmons and Fire, 1998). It is now understood that RNAi can be achieved through two distinct but homologous pathways, both of which are widely conserved. The first is an immune response that is triggered by the presence of long dsRNA molecules (>60 bp in length), which likely resemble viral nucleic acids. The end result of dsRNA processing by a Dicer enzyme is a “pool” of siRNAs that represent the entire length of the dsRNA. While the abundance of any single siRNA will be low, due to the stochastic nature of the processing, multiple identical sequences match to the original dsRNA, or any subsequent transcripts that might be produced from it. The second pathway is used by endogenous micro RNAs (miRNAs). These small RNA transcripts contain inverted repeat sequences that form one or more stem-loop structures where the stem consists of dsRNA and the loop is unpaired, single-stranded RNA. These primary miRNAs are then processed by a Dicer enzyme into mature miRNAs that are approximately 22 bp in length and function as siRNAs. Because of the size and structure of the primary miRNA transcript and the specificity of the processing, the result is a single siRNA population which may target multiple transcripts (Siomi and Siomi, 2010).

These discoveries had a profound impact on our understanding of gene regulation, and provided a powerful tool for conducting research into gene function. It is not an exaggeration to suggest that the ability to employ a sequence specific, inducible gene silencing mechanism coupled with the rapidly expanding genomic resources has reshaped biological research in the first decade and a half of the twenty-first century. The implications of dietary RNAi, also termed environmental RNAi or eRNAi, were quickly recognized by researchers interested in human therapeutics (Lares et al., 2011; Witwer and Hirschi, 2014; Hirschi et al., 2015) and in plant protection (Baum et al., 2007; Mao et al., 2007; Burand and Hunter, 2013; Koch and Kogel, 2014). The potential to use the technology for pest control through the expression of dsRNA in genetically engineered (GE) plants, *in planta* RNAi, has led to discussions of how best to collect data for informing an ecological risk assessment, with a particular focus on the effects on non-target organisms (NTOs) representing diverse ecological functions, including natural enemies, pollinators, soil decomposers, leaf shredders, wildlife, and fish (Auer and Frederick, 2009; Center for Environmental Risk Assessment, 2011; Bachman et al., 2013; Lundgren and Duan, 2013; Ramesh, 2013; European Food Safety Authority, 2014; Ramon et al., 2014; US EPA, 2014; Casacuberta et al., 2015).

This publication is intended to serve as a resource, providing a review of current knowledge about the mechanism and susceptibility of NTOs to eRNAi, and making observations about areas of research that may provide value or improved certainty to future risk assessments of RNAi-based GE plants. It builds upon presentations made at the 13th International Symposium on Biosafety of Genetically Modified Plants (South Africa, Cape Town, 9–13 November 2014), and is focused on the use of RNAi in GE plants. Issues surrounding the regulation of RNAi-based GE plants, data requirements, and standards for case by case risk assessment of any particular GE plant will not be discussed. Nothing in this paper is intended to suggest that the identified areas of potentially beneficial research are a pre-requisite for the completion of a scientifically sound, case specific environmental risk assessment for an RNAi-based GE plant. However, basic research advancing our understanding of RNAi mechanisms and how NTOs respond to eRNAi could provide value to future assessments of RNAi-based biotechnologies.

## Pathway to harm for NTOs

**Figure 1:**
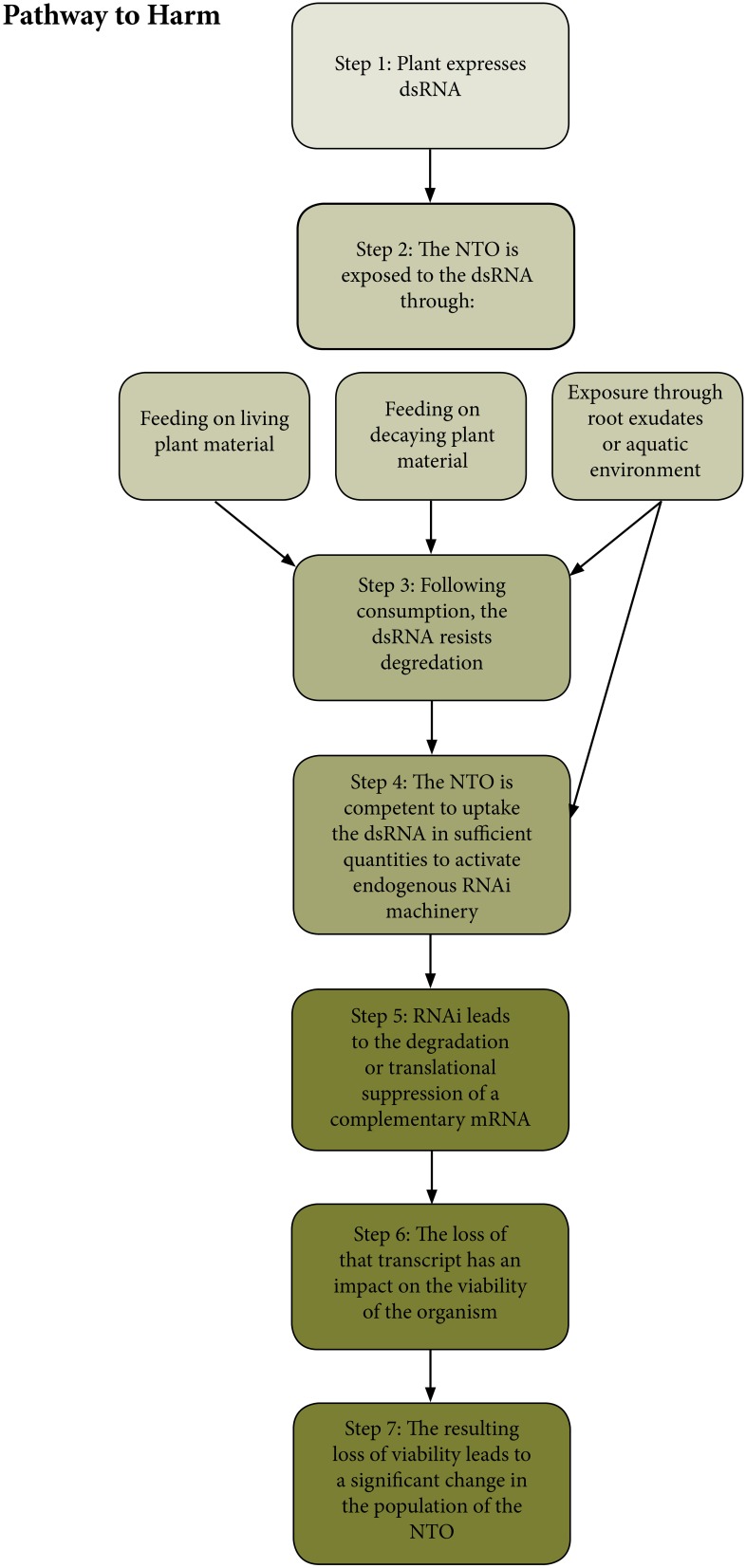
**A diagram representing the hypothetical pathway to harm for non-target organisms exposed to dsRNA produced in a plant**. This diagram considers only primary exposure, rather than multi-trophic interactions because primary exposures are considered by the authors to be the most plausible pathway to harm relevant to dsRNA exposure.

In order to identify areas where additional research might prove valuable for informing future risk assessments of RNAi-based GE plants, it is first necessary to envision a plausible pathway to harm whereby NTOs might be exposed to dsRNA from a plant, leading to an adverse environmental consequence (Raybould, 2006; Wolt et al., 2010; Gray, 2012). The pathway to harm explains how the deployment of the GE plant could lead to adverse impacts on NTOs through a chain of events taking account of both hazard and exposure. In addition to conceptualizing the relationship between the plant and NTOs, it is also useful for identifying which steps in the pathway can be most easily investigated through testing. First, the plants must be expressing a dsRNA. Then NTOs must be exposed to that dsRNA. The greatest exposure would be expected to occur to NTOs feeding directly on living plant material although exposure from consumption of pollen, cuttings, leaf litter, or other plant materials or exudates into soil or aquatic environment is also a possibility, provided the dsRNA persists in that environment at sufficient concentrations. Following consumption the dsRNA must resist degradation in the gut. The NTO must be competent to uptake the dsRNA in sufficient quantities to activate its endogenous RNAi machinery. This can occur either locally at the point of uptake (e.g., in cells lining the gut), or systemically if the NTO is capable of triggering systemic RNAi (Smagghe and Swevers, 2014; Ivashuta et al., 2015). Once the endogenous RNAi machinery is active, it must lead to the degradation or translational suppression of a corresponding mRNA in a sequence dependent fashion (Whyard et al., 2009; Zotti and Smagghe, 2015). And finally, the loss of that transcript must have an adverse impact on the NTO (Bachman et al., 2013). A diagram illustrating the generalized pathways to harm for RNAi expressing GE plants is found in Figure 1.

If any of these steps is unlikely or impossible, then the risk to a NTO from an RNAi-based GE plant is negligible. Some elements of this generic pathway will be case specific—for example the sequence of a particular dsRNA will determine whether or not an mRNA with a complementary sequence is available for silencing. However, there are steps in this pathway that will be common to most or all cases, and these represent potential targets for conducting basic research, so as to gather baseline data in support of the risk assessment of RNAi-based GM plants. These include the inherent susceptibility of a NTO to dsRNA in the environment, as well as the ability of dsRNA to persist in the environment, and finally the potential for inducing off-target gene silencing in a NTO.

## What makes an organism susceptible to dsRNA in the environment?

For researchers, particularly those working with *C. elegans* as a model organism, the discovery that animals could be exposed to dsRNA through feeding and show a sequence specific gene knockdown effect transformed the field of RNAi research. Even before the mechanism was well understood, and before a thorough understanding of the role of RNAi as a regulator of development, the technique was rapidly adopted and reverse genetic techniques quickly supplanted traditional forward genetic screens. Within 5 years of the seminal publication by Fire et al., a collection of *E. coli* containing dsRNA constructs was developed and made available to the research community, making it possible to conduct phenotypic assays by feeding *C. elegans* with these GE bacteria (Kamath and Ahringer, 2003; Kamath et al., 2003). Once the phenotype was observed, a researcher could simply look up the gene corresponding to the dsRNA being expressed by the bacteria. However, it soon became clear that an element of serendipity had facilitated the use of RNAi in *C. elegans*. *Caenorhabditis briggsae*, a congeneric species of *C. elegans*, is not responsive to environmental dsRNA (Winston et al., 2007; Whangbo and Hunter, 2008). These two nematode species are morphologically indistinguishable, occupying the same ecological niche, and sharing striking genetic similarity (Stein et al., 2003). This difference is due to differences in a single gene, *sid-2*, which encodes a conserved transmembrane protein localized to the intestinal lumen. Transfection of *C. briggsae* with *C. elegans sid-2* can confer susceptibility to dsRNA in the environment and further study revealed that susceptibility is rare in the genus Caenorhabditis—present in only one other known species that is not closely related to *C. elegans* (Winston et al., 2007). With regard to RNAi, the only known difference between *C. elegans* and *C. briggsae* is the ability to uptake dsRNA from the environment, as both organisms possess the capacity for cell autonomous and systemic RNAi, in which the silencing signal is spread from cell to cell or from one part of an organism to another (Winston et al., 2007).

Experience with the use of RNAi in other model organisms suggests that nematodes are not unique in this regard, and the ability to respond to dsRNA in the environment shows tremendous variability—sometimes between closely related organisms. In invertebrates, susceptibility to dsRNA has been observed in many species, including cnidarians (Hydra), planaria (flatworms), and various arthropods (Zotti and Smagghe, 2015). Interestingly, it also appears that environmental susceptibility is common in parasitic nematodes (Caenorhabditis species are soil dwelling, and not parasitic; Whangbo and Hunter, 2008).

Future research to improve our understanding of how and why organisms are susceptible or unresponsive to environment RNAi may help provide a rationale for choosing test organisms for NTO studies, as well as identifying species which should be given particular attention and species which do not need to be considered in assessments.

### Environmental RNAi in arthropods

Among the invertebrates, arthropods can be major pests of plants or provide valuable ecological and agricultural services. Although much work is being done on understanding RNAi in other invertebrates, we will focus our discussion on arthropods (and particularly insects) because these are likely to be the target pests for RNAi-based GE plants. The use of plant expressed dsRNA to confer resistance to insects was among the earliest non-research applications to be widely considered, together with use for human therapies, and the first demonstration of its effectiveness was reported in 2007 (Baum et al., 2007; Mao et al., 2007). Following these publications, and others advancing the concept (Baum et al., 2007; Bolognesi et al., 2012; Rangasamy and Siegfried, 2012), the consideration of the needs and modalities for environmental risk assessment related to insect resistant plants making use of RNAi have been widespread (Auer and Frederick, 2009; Center for Environmental Risk Assessment, 2011; Lundgren and Duan, 2013; Ramon et al., 2014; Casacuberta et al., 2015).

Arthropods display a wide range of sensitivities to ingested dsRNA (Bellés, 2010), with coleopterans showing significantly greater sensitivity than other arthropod orders. Lepidopteran species have variable susceptibility to ingested dsRNA and require high concentrations of dsRNA to elicit a response comparable to coleopterans (Terenius et al., 2011; Ivashuta et al., 2015). The use of RNAi as a tool for research in insects has been extensive, although the utility has not been as robust as in *C. elegans.* For example, *Drosophila melanogaster* only shows a response to dsRNA at the site of delivery through microinjection, and the effect is transient (Price and Gatehouse, 2008). Drosophila are not susceptible to environmental dsRNA through soaking or feeding, although this can be overcome with the assistance of chemical enhancers (Whyard et al., 2009). Other insects vary widely in their ability to respond to environmental dsRNA as well as their ability to elicit systemic RNAi through microinjection or other means (Bellés, 2010; Huvenne and Smagghe, 2010; Terenius et al., 2011; Gu and Knipple, 2013). This despite possessing all of the necessary cellular machinery and clearly functional cell autonomous RNAi. The correlation between the *C. elegans* genes identified as important for dsRNA uptake with homologs in susceptible insects is low, as is the correlation between homologs for systemic RNAi (Huvenne and Smagghe, 2010). This suggests that even when insects are susceptible to environmental RNAi, the mechanism is not the same (Huvenne and Smagghe, 2010). Further, the reluctance to publish negative results likely leads to an overestimation of the general susceptibility of insects to RNAi techniques (Bellés, 2010). However, some systematic studies are attempting to collect and organize disparate reports on experiences with RNAi in insects, including an effort to curate a database of experiments involving lepidopterans (Terenius et al., 2011; Christiaens and Smagghe, 2014; Kolliopoulou and Swevers, 2014).

Multiple factors can affect RNAi efficiency in insects, including dsRNA concentrations, lengths of dsRNA fragments, the timing and duration of exposure, dsRNA uptake and degradation activities, activation of RNAi machinery, and the life stage of the target organisms (Huvenne and Smagghe, 2010; Terenius et al., 2011; Bolognesi et al., 2012; Chu et al., 2014; Coleman et al., 2014; Ivashuta et al., 2015). Information on barriers to exposure such as the potential degradation of dsRNA prior to ingestion, barriers to cellular uptake, instability of the dsRNA within the recipient organism following ingestion, and the inherent sensitivity of the organism to ingested dsRNA could facilitate risk assessment predictions across non-target taxa, refine exposure estimates, or allow assumptions of minimal exposure in certain organisms. At present, however, there is insufficient understanding on specific barriers to make any generalizations (Ramon et al., 2014; US EPA, 2014). An improved understanding of how and why susceptible insects take up dsRNA from the environment, as well as a more complete picture of what insect orders possess the capacity for systemic RNAi will be informative for identifying species that may require consideration during risk assessment, or species that can be eliminated from consideration due to their inability to respond to environmental RNAi.

### Environmental RNAi in vertebrates

Studies of vertebrate RNAi have been centered around the development of RNA based therapeutics. Many of these focus on introducing dsRNA through direct injection or transfection, and reports of oral delivery suggest that carriers or other protective delivery mechanisms are required to protect the RNA from degradation in the digestive system. Based on anecdotal and unpublished information from clinical trials, the delivery of naked RNA through mammalian digestive tract is not likely (Witwer and Hirschi, 2014). However, Zhang and colleagues reported that miRNAs from plant-based foods can be detected in human and mouse tissue and regulate gene expression (Zhang et al., 2012). This report generated significant attention from both the scientific and popular media because of implications for diet and nutrition, potential use in therapeutics as well as implications for possible hazards associated with consumption of RNAi-based GE plants. While some researchers reported data consistent with the original study (Wang et al., 2012; Beatty et al., 2014), others have failed to replicate the results or verify them through alternative means (Dickinson et al., 2013; Snow et al., 2013; Witwer et al., 2013; Witwer and Hirschi, 2014). This, coupled with observations about the copy number required to elicit RNAi in human cells and the quantity of plant miRNA required, has led to a consensus that if plant miRNAs are present in mammalian serum, they are at insufficient quantities to regulate gene expression. Nevertheless, the research group reporting the initial finding has responded to some of these reports (Chen et al., 2013), and has published additional work detailing the detection of other plant derived miRNAs in humans and mice after feeding, with biological activity (Zhou et al., 2014; Liang et al., 2015). These results would benefit from validation by other research groups, and hypothesize the existence of a specific mechanism for transporting exogenous miRNA from the gut to target tissues within mammals where they are capable of regulating mammalian genes. Most recently, Mlotshwa et al. (2015) treated a mouse model of intestinal cancer with a high-dose cocktail of synthetic versions of three miRNAs reported to be deficient in some tumor cells. The authors reported that mice exposed to the regimen had a reduced tumor burden, hypothesizing that the synthetic RNAs had been taken up by cells and were limiting expression of tumor-related genes. As discussed at the ISBGMO 2014 meeting, no data were provided on whether the RNAs had been taken up by cells in a functional form, and surface interaction effects of high-dose RNA could not be excluded (Mlotshwa et al., 2015). If validated, this represents a pathway of exposure to plant produced miRNAs in mammals (Hirschi et al., 2015).

It is important to note that, just like for invertebrates, NTO assessments for mammals and other vertebrates consider whether exposure to a specific RNA would lead to harm. The research described above is concerned with only one element, exposure. In order for an exogenous dsRNA expressed in a plant to harm a vertebrate, a series of events would have to occur which include the consumption of plant materials containing the dsRNA, the survival of dsRNA in the digestive track and uptake of dsRNA, followed by the delivery to a target tissue or tissues in sufficient quantity to activate RNAi. In order to activate RNAi, there would also need to be sufficient sequence complementarity with an mRNA transcript in the target cells (Jensen et al., 2013). Finally, the silencing of gene expression would need to result in harmful effects on the organism. The likelihood of this series of events would be considered on a case by case basis for the particular dsRNA-expressing plant being assessed and for the NTOs being considered.

Additional studies which bring clarity to the ability of mammalian or other vertebrate organisms to take up dsRNA from the environment, as well as on the dietary parameters affecting uptake, would certainly provide value to future risk assessments (Hirschi et al., 2015). In particular, many of the above studies focus on identifying the presence or absence of RNA sequences in tissue or sera, and linking that presence to measurements of biological activity which may be ambiguous or confounded by other factors. Hirschi et al. (2015) stressed that dietary regimens involving small dosages over a short period of time simply may not be sufficient to result in measurable uptake, and that the failure to detect the plant-derived small RNAs in the serum of mammals may not reflect failure of absorption, but rather failure to account for the subsequent metabolism, distribution, and elimination of the small RNAs. Therefore, experiments designed to provide simple and unambiguous results are highly recommended. One example might be the delivery of dsRNA with sequence complementarity to a reporter gene (such as Green Fluorescent Protein—GFP) into the digestive system of a mouse expressing that reporter in specific tissues to determine if gene suppression can be achieved. Another possibility would be a similar experiment targeting an essential gene. The authors fully recognize that while these experiments are simple to suggest, they are expensive and complicated to conduct. However, underpinning much of the conversation surrounding mammalian uptake of dsRNA is a widespread anecdotal belief that such “proof of concept” studies have already been conducted by researchers looking into therapeutic applications for RNAi. If such studies exist, then publication of these negative results would be highly beneficial to resolve lingering uncertainties.

## Understanding the potential for off-target gene effects

For the purposes of this discussion, an “off-target gene effect” refers to any gene being silenced that is not the intended target, either in the organisms producing the dsRNA or, more relevant to NTO risk assessment, in an organism exposed to the dsRNA that is not the intended target organism. It is known that multiple stretches of sequence homology on a long dsRNA can increase the efficacy of RNAi silencing in insects (Whyard et al., 2009). However, although effective RNAi with sequence mismatch has not been shown in insects, it has been observed in plants and human cell lines that even with some level of sequence mismatch to a processed 21–25 bp siRNAs, silencing may still occur (Du, 2005; Liu et al., 2014). The “forgiveness” of mismatches could increase the potential for off-target gene silencing effects. Moreover, some siRNAs resulting from the cleavage of a dsRNA by Dicer or its homologs appear to persist at a higher concentration than others (i.e., processing may result in non-random selection of siRNAs). It seems apparent that an improved understanding of how siRNAs are processed and maintained, incorporated into the RISC and what level of base pair mismatch can be tolerated might improve our understanding of how to assess the likelihood of impacts to NTOs. It is worth mentioning, however, that off-target gene effects can only occur in organisms with competency for uptake from the environment (presumably through the gut), and exposure to the dsRNA in sufficient quantities to allow RNAi in either a cell autonomous fashion in the gut epithelial cells or in a systemic RNAi response in other tissues. Only then will sequence specificity come into play, so in this sense, information regarding sequence specific off-target gene effects represents the penultimate step in the pathway to harm (followed only by the gene specific silencing leading to an effect which may or may not be harmful). However, in cases where an NTO is known to be susceptible, and is predicted to be exposed to the dsRNA in the environment in sufficient quantities (Romeis et al., 2014), it may be useful to understand how likely off-target gene effects are to be realized.

There are two ways to approach this question related to NTOs, and there are examples of both approaches in the current literature (Whyard et al., 2009; Bolognesi et al., 2012; Bachman et al., 2013). The first is to look at the basic biology of the RNAi machinery and try to determine how well a sequence needs to match in order to ellicit an effect. This is a bioinformatics-based approach. Bioinformatic analyses of sequence complementarity between the pool of siRNAs and the target gene could help to identify potential off-target genes in NTOs. This approach could guide the selection of non-target species harboring genes that share a certain level of homology with the target gene in the pest, and thus those species should be the focus of further assessment. Moreover, if reliable bioinformatic data indicate that the minimum sequence requirements for RNAi activity are not met between non-target and target species, then further assessment may not be necessary, as the likelihood of adverse effects is low. However, this approach is currently subject to substantial limitations (European Food Safety Authority, 2014; Ramon et al., 2014; US EPA, 2014; Casacuberta et al., 2015), which have implications for the prediction of potential off-target gene effects, and the usability of bioinformatic data in support of the environmental risk assessment. The bioinformatics-based approach requires knowledge of sequence information, which may not be available for all species of interest. It may also be subject to differences between organisms in terms of how the RNAi machinery functions in relation to base pair mismatches. Moreover, scientific uncertainties remain on the exact rules governing small RNA-mRNA matches/interactions. Hence, progress of basic research on RNAi mechanisms, production of suitable genome data for relevant species, and design of efficient algorithms to make reliable predictions will increase the usability of bioinformatic data in support of environmental risk assessments of RNAi-based GM plants.

The other way to approach the problem is to introduce dsRNA that is perfectly homologous to the target gene in a target organism, to a range of other organisms, starting with close relatives, and then moving outward to see how phylogenetically distant organisms respond (Bachman et al., 2013). This approach enables characterization of the activity spectrum of dsRNAs, and can be done without sequence information from the tested species. Bachman et al. (2013) suggest that, in tested insects, close phylogenetic relationships are required for off-target gene effects—provided the target gene selected is not highly conserved, and that at least one sequence match of greater than 19 bp to the target sequence is necessary to see significant activity. Currently, standardized protocols to evaluate the potential hazards of RNAi-based GE plants to NTOs are under development and risk hypotheses are being tested using early-tier assessment methods (http://nifa.usda.gov/sites/default/files/resource/brag_pd_mtg_2014_0.pdf). The experience gained will indicate whether NTO bioassays are appropriately attuned to assess the effects of RNAi on the fitness and performance of NTOs. The timing and duration of exposure necessary to achieve the RNAi response are uncertain, and a more thorough investigation of dose-response relationships for siRNA targets would therefore be necessary for RNAi susceptible NTOs. Since the usefulness of NTO bioassays with plant material to capture unknown complexities and variability in the RNAi-based GE plant remains a contentious point of debate (US EPA, 2014; Devos et al., 2015), it would be helpful to investigate whether such bioassays will add weight of evidence to the NTO risk assessment, and what determines their need.

## Persistence of dsRNA in the environment

The primary route of exposure for an organism to encounter dsRNA from a GE plant is expected to be oral ingestion of living plant material (US EPA, 2014). However, an understanding of the environmental fate of RNAi molecules may be informative in ruling out other potential exposures. For plant material in an agricultural setting, the persistence of dsRNA in leaf litter or the soil is an important potential route of exposure. The authors are not aware of any data on the persistence of dsRNA in leaf litter, however, experimental evidence suggests that dsRNA breaks down quickly in soil (90% degradation by 35 h, Dubelman et al., 2014). Although adsorption to soil particles cannot be ruled out, insects challenged with a soil matrix containing an excessive amount of dsRNAs that far exceeded the concentration in GE plants did not elicit an RNAi response (Dubelman et al., 2014).

The other potential source of exposure is through food webs with dsRNA potentially being transferred to different trophic levels. The theoretical pathway to harm involves the uptake of dsRNA by an organism serving as prey for predators or host for parasitoids, thus exposing natural enemies to the dsRNA when feeding on or parasitizing their prey or host, respectively. However, at least for insects susceptible to eRNAi, experiments suggest that the long dsRNA rather than siRNA is necessary to elicit a response (Bolognesi et al., 2012; Ivashuta et al., 2015; Li et al., 2015). While only long dsRNA can be efficiently taken up from gut lumen, evidence suggests that both long dsRNAs and processed siRNAs are transported to other insect organs and tissues (Ivashuta et al., 2015). Therefore, the primary consumer (prey/host) would have to consume dsRNA and then preserve it without processing it into siRNA, or somehow amplify the dsRNA in order to expose secondary or tertiary consumers (natural enemies) to sufficient quantities to elicit a persistent response. There is no evidence for the existence of mechanisms for either of these processes, although they have not been the subject of thorough investigation. On-going research is currently establishing which species are at risk through consuming dsRNA-containing maize tissue under field conditions, and whether dsRNAs are transferred to higher trophic levels via consuming herbivorous prey. The gathered data will help to predict which species are exposed to dsRNAs and which of those are at risk of off-target gene effects of RNAi molecules^1^.

For both the persistence of dsRNA in the soil and leaf litter as well as the potential for eliciting RNAi through the food web, it may be possible to address these scenarios through a number of well-designed experiments rather than requiring the collection of case specific data for each and every future application of RNAi in a GE plant. Soil studies looking at dsRNA of different lengths and base pair composition, as well as secondary structures should provide insight into whether any significant variation in degradation time is expected. Similarly, experiments with primary consumers could be done which identify any potential for the amplification or transmission of RNAi through the food web.

## Conclusion

The discovery of RNAi and its application to biological research has profound impacts on our knowledge of gene regulation and the way we conduct research. It also has the potential for practical applications in producing desirable phenotypes in plants either through the selective silencing of target genes in the plant or through the production of dsRNA complementary to mRNA transcripts in target pests. The use of this technology, particularly through GE plants, will be accompanied by environmental risk assessments which will consider the potential for harmful impacts to NTOs. Although current knowledge may well be sufficient to conduct case specific risk assessments, it is clear that our current understanding of the susceptibility of organisms to environmental exposure to dsRNA, as well as the parameters which influence the likelihood of off-target gene effects are not complete. Additional research addressing these areas is warranted to improve the certainty associated with risk assessments of RNAi applications, and contribute to reducing the burden of case specific data collection and testing. Finally, the publication of negative results which have implications for the susceptibility of an organism to eRNAi, from both invertebrate and vertebrate research, could greatly contribute to our overall understanding of what NTOs have the potential to be affected by the use of dsRNA in the environment.

## Author note

All authors participated in the drafting of this paper as individual experts in their fields, and the authors are solely responsible for the contents. Any views expressed in this paper are the views of the authors and do not necessarily represent the views of any organization, institution, or government with which they are affiliated or employed.

### Conflict of interest statement

The authors declare that the research was conducted in the absence of any commercial or financial relationships that could be construed as a potential conflict of interest.

## Footnotes

^1^http://nifa.usda.gov/sites/default/files/resource/brag_pd_mtg_2014_0.pdf
